# Unsupervised grammar induction of clinical report sublanguage

**DOI:** 10.1186/2041-1480-3-S3-S4

**Published:** 2012-10-05

**Authors:** Rohit J Kate

**Affiliations:** 1 Department of Health Informatics and Administration, Department of Computer Science, University of Wisconsin-Milwaukee, Milwaukee, WI 53211, USA

## Abstract

**Background:**

Clinical reports are written using a subset of natural language while employing many domain-specific terms; such a language is also known as a sublanguage for a scientific or a technical domain. Different genres of clinical reports use different sublaguages, and in addition, different medical facilities use different medical language conventions. This makes supervised training of a parser for clinical sentences very difficult as it would require expensive annotation effort to adapt to every type of clinical text.

**Methods:**

In this paper, we present an unsupervised method which automatically induces a grammar and a parser for the sublanguage of a given genre of clinical reports from a corpus with no annotations. In order to capture sentence structures specific to clinical domains, the grammar is induced in terms of semantic classes of clinical terms in addition to part-of-speech tags. Our method induces grammar by minimizing the combined encoding cost of the grammar and the corresponding sentence derivations. The probabilities for the productions of the induced grammar are then learned from the unannotated corpus using an instance of the expectation-maximization algorithm.

**Results:**

Our experiments show that the induced grammar is able to parse novel sentences. Using a dataset of discharge summary sentences with no annotations, our method obtains 60.5% F-measure for parse-bracketing on sentences of maximum length 10. By varying a parameter, the method can induce a range of grammars, from very specific to very general, and obtains the best performance in between the two extremes.

## Introduction

Obtaining a syntactic parse is an important step in analyzing a sentence. Syntactic parsers are typically built using supervised learning methods. Several hundred or thousand sentences are first manually annotated with syntactic parses. Then some learning method is employed that learns from this annotated data how to parse novel sentences. A major drawback of this approach is that it requires a lot of manual effort from trained linguists to annotate sentences. Also, a parser trained in one domain does not do well on another domain without adapting it with extra annotations from the new domain. This drawback becomes even more severe when the domain is that of clinical reports or medical text, because the annotators need to be not only trained linguists but also need to have sufficient clinical knowledge to understand the clinical terms and the sentence forms. This is a rare combination of expertise, which makes the annotation process for clinical reports even more expensive. Also, different genres of clinical reports, like discharge summaries, radiology notes, cardiology reports etc., are different from each other and hence will require separate annotations. On top of that, different hospitals or medical centers may be using their own convention of clinical terms and sentence styles in writing clinical reports which may require separate annotation effort to adapt a syntactic parser to work for clinical reports across institutions.

Besides the annotation effort required, another drawback of supervised syntactic parsing is that it forces a particular "gold standard" of syntactic parses which may not be best suited for the end-application in which the syntactic parses will be used. For example, in the application of semantic parsing, the task of converting a sentence into an executable meaning representation, it was found that the conventional gold standard syntactic parse trees were not always isomorphic with their semantic trees [1] which lowered the performance of semantic parsing. In the domain of clinical reports, where sentences are often succinct and may not follow typical English grammar, it is not easy to decide the gold standard parses in advance. For example, a sentence like "Vitamin B12 250 mcg daily" could be parsed with brackets such as "((Vitamin B12 250 mcg) daily)" or such as "((Vitamin B12) (250 mcg daily))" depending upon whether the end-application emphasizes the "250 mcg" quantity with "Vitamin B12" or with "daily". However, during the annotation process, a particular form will get forced as part of the annotation convention without regards to what may be better suited for the end-application down the road. Syntactic parses are not an end in themselves but an intermediate form which is supposed to help an end-application, hence it will be best if such an intermediate form is not set in advance but gets decided based on the end-application. An alternate to supervised learning for building parsers is unsupervised learning. In this framework, a large set of unannotated sentences, which are often easily obtainable, are given to an unsupervised learning method. Using some criteria or bias, for example, simplicity of grammar and corresponding sentence derivations, the method tries to induce a grammar that best fits all the sentences. Novel sentences are then parsed using this learned grammar. While unsupervised parsing methods are not as accurate as supervised methods, their no demand of manual supervision makes them an attractive alternative, especially for the domain of clinical reports for the reasons pointed out earlier.

An additional advantage of unsupervised parsing is that the grammar induction process itself may be adapted so as to do best on the end-application. For example, instead of using a simplicity bias to guide the grammar induction process, a criterion to maximize accuracy on the end-application may be used. This way the induced grammar may choose one parse over another for the "Vitamin B12 250 mcg daily" depending upon which way is more helpful for the end-application. In [2], an analogous approach was used to transform a semantic grammar to best suit the semantic parsing application.

In this paper, we present an approach for unsupervised grammar induction for clinical reports, which to our knowledge is the first such attempt. We adapt and extend the simplicity bias (or cost reduction) method [3] of unsupervised grammar induction. We chose to use this method because its iterative grammar-modifying process using grammar transformation operators is amenable for adaptation to any criterion besides simplicity bias. This could be useful for adapting the grammar induction process to maximally benefit some end-application. Another advantage of this method is that it directly gives the grammar in terms of non-terminals it creates on its own; some other existing methods only give bracketing [4,5] or force the user to specify the number of non-terminals [6]. The induced grammar is also not restricted to binary form unlike in some previous methods [6,7]. After inducing the grammar, in order to do statistical parsing, the probabilities for its production are obtained using an instance of the expectation-maximization (EM) algorithm [8] run over the unannotated training sentences.

**Figure 1:**
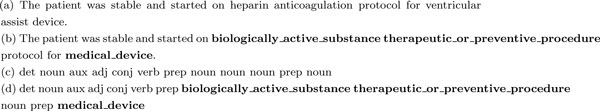
**(a) An original sentence, (b) some of its words replaced by UMLS semantic types (bold), (c) its words replaced by part-of-speech tags, (d) its words replaced by part-of-speech tags and UMLS semantic types (bold) wherever applicable**.

We used sentences from discharge summaries of the Pittsburgh corpus [9] as our unannotated data. Most unsupervised grammar induction methods work with part-of-speech tags because large vocabularies make it difficult to directly induce grammars using the words themselves. Since the language used in clinical reports is a domain-specific sublanguage [10-12], it uses several terms, like disease names, medications etc., not generally found in normal language. This makes the vocabulary even larger. We also note that for parsing clinical reports, besides using part-of-speech tags, it will be a good idea to also use semantic classes of words because they often affect syntactic structure of a sentence. Hence we decided to also utilize UMLS semantic types [13] of the clinical terms (for example, disease, sign or symptom, finding etc.), which, in a way, are treated like additional part-of-speech tags in the grammar induction process (Figure 1(d) shows an example). These semantic types and the part-of-speech tags are obtained using MetaMap [14]. The grammar is then learned in terms of part-of-speech tags and the semantic types of clinical terms.

In the experiments, we first show that the learned grammar is able to parse novel sentences. Measuring accuracy of parses obtained through an unsupervised parsing method is always challenging, because the parses obtained by the unsupervised method may be good in some way even though they may not match the correct parses. The ideal way to measure the performance of unsupervised parsing is to measure how well it helps in an end-application. However, at present, in order to measure the parsing accuracy, we annotated one hundred sentences with parsing brackets and measured how well they match against the brackets obtained when parsed with the induced grammar.

## Cost reduction method for grammar induction

For inducing a context-free grammar from training sentences, we adapted the cost reduction method [3] which was based on Wolff's idea of language and data compression [15], also known as simplicity bias method or minimum description length method. The method starts with a large trivial grammar which has a separate production corresponding to each training sentence. It then heuristically searches for a smaller grammar as well as simpler sentence derivations by repeatedly applying grammar transformation operators of combining and merging non-terminals. The size of the grammar and derivations is measured in terms of their encoding cost. We have extended this method in a few ways. We describe the method and our extensions in this section. We first describe how the cost is computed in Subsection and then describe the search procedure that searches for the grammar that leads to the minimum cost in Subsection. In Subsection, we describe how the probabilities associated with the productions of the induced grammar are computed.

### Computing the cost

The method uses ideas from information theory and views the grammar as a means to compress the description of the given set of unannotated training sentences. It measures the compression in terms of two types of costs. The first is the cost (in bits) of encoding the grammar itself. The second is the cost of encoding the sentence derivations using that grammar. In the following description we make use of some of the notations from [16].

#### Cost of grammar

A production in a context-free grammar (CFG) is written in the form of *A *→ *β*, where *A *is a non-terminal and *β *is a non-empty sequence of terminals and non-terminals. The cost, *C_P_*, of encoding this production is:

(1) $$C_{P}=\left(1+|\beta |\right)log|\Sigma |$$

where |*β*| is the length of the right-hand-side (RHS) of the production, and |Σ| is the number of terminals and non-terminals in the symbol set Σ. Since it will take *log*|Σ| bits to encode each symbol and there are (1 + |*β*|) symbols in the production (including left-hand-side (LHS)), hence the cost *C_P _*of encoding the production is as given in the above equation. Thus the cost of encoding the entire grammar, *C_G_*, is:

(2) $$C_{G}=\overset{p}{\underset{i=1}{\sum }}\left(1+|\beta_{i}|\right)log|\Sigma |)$$

where *p *is the number of productions and *β_i _*is the RHS of the *i*th production.

#### Cost of derivations

Given the grammar, a derivation of a sentence proceeds by first expanding the start symbol of the grammar with an appropriate production and then subsequently recursively expanding each of the RHS non-terminals until all the symbols of the sentence are found as a sequence of terminals. At every step in the derivation process, an appropriate production needs to be selected to expand a non-terminal. This is the only information that needs to be encoded in order to encode the sentence. Hence the information to be encoded at every step of the derivation is: which of the |*P*(*s_k_*)| productions was used to expand the *k*th non-terminal, *s_k_*, in the derivation process, *P*(*s_k_*) being the set of productions in which *s_k _*is the LHS. This information can be encoded in *log*(|*P*(*s_k_*)|) bits. For example, if there is only one way to expand a non-terminal then this information is obvious and would require zero bits to encode. Hence the cost of an entire derivation, $C_{D_{j}}$ of the *j*th sentence will be:

(3) $${C_{D}}_{{}_{j}}=\Sigma_{k=1}^{m_{j}}\left(log\left(\left|P\left(s_{k}\right)\right|\right)\right)$$

where *m_j _*is the length of derivation of the *j*th sentence. Thus the cost, *C_D_*, of encoding all *q *sentences in the training set is:

(4) $$C_{D}=\Sigma_{j=1}^{q}\overset{m_{j}}{\underset{k=1}{\sum }}\left(log\left(\left|P\left(s_{k}\right)\right|\right)\right)$$

#### Total cost

In previous work, like [3] and [16], the total cost of grammar and derivation was taken as simply the sum of the individual costs. However, as we show in the experiments, this does not always lead to good results. The reason, we believe, is that the total cost of derivations depends on the number of sentences and simply adding this cost to the grammar's cost may lead to an unequal weighting. To remedy this, we introduce a parameter, *f*, that takes values between 0 and 1, to separately weigh the two components of the total weight *C *as follows:

(5) $$C=f*C_{G}+\left(1-f\right)*C_{D}$$

where *C_G _*is the cost of the grammar and *C_D _*is the cost of all derivations as described before. Note that *f *= 0.5 is equivalent to adding the two components as in the previous work. In the experiments, we vary this parameter and empirically measure the performance.

### Grammar search for minimum cost

It is important to point out that there is a trade-off between the cost of the grammar and the cost of the derivations. At one extreme is a simplest grammar in which there are productions like *NT *→ *t_i_*, i.e. a non-terminal *NT *that expands to every terminal *t_i_*, and two more productions *S *→ *NT *and *S *→ *SS*, (*S *being the start symbol) which will have a very little cost. However, this grammar will lead to very long and expensive derivations. It is also worth pointing out that this grammar is overly general and will parse any sequence of terminals.

On the other extreme is a grammar in which each production encodes an entire sentence from the training set, for example, *S *→ *w*_1_*w*_2_..*w_n_*, where *w*_1_, *w*_2 _etc. are words of a sentence. The derivations of this grammar will have very little cost, however, the grammar will be very expensive as it will have long productions and as many of them as the number of sentences. It is also worth pointing out that this grammar is overly specific and will not parse any other sentence besides the ones in the training set. Hence the best grammar lies in between the two extremes, which will be general enough to parse novel sentences but at the same time not too general to parse almost any sequence of terminals. This grammar will also have a smaller cost than either extreme. According to the minimum description length principle as well as Occam's razor principle, a grammar with minimum cost is likely to have the best generalization. We use the following search procedure to find the grammar which gives the minimum total cost where the total cost is as defined in equation 5. We note that by varying the value of the parameter *f *in that definition, the minimum cost search procedure can find different extremes of the grammars. For example, with *f *= 1, it will find the first type of extreme grammar with the least grammar cost, and with *f *= 0, it will find the second type of extreme grammar with the least derivation cost.

The search procedure begins with a trivial grammar which is similar to the second extreme type of grammar mentioned before. A separate production is included for each unique sentence in the training data. If the sentence is *w*_1_*w*_2_..*w_n_*, a production *S *→ *W*_1_*W*_2_..*W_n _*is included along with productions *W*_1 _→ *w*_1_, *W*_2 _→ *w*_2_, etc., where *W*_1_, *W*_2_, etc. are new non-terminals corresponding to the respective terminals *w*_1_, *w*_2_, etc. The new non-terminals are introduced because the grammar transformation operators described below do not directly work with terminals. Instances of the two grammar transformation operators described below are then applied in a sequence in a greedy manner, each time reducing the total cost. We first describe the two operators, *combine *and *merge*, and then describe the greedy procedure that applies them. While the *merge *operator is same as in [3], we have generalized the *combine *operator (which they called *create *operator). The search procedure is analogous to theirs but we first efficiently estimate the reductions in cost obtained by different instances of the operators and then apply the one which gives the most reduction in cost. They on the other hand do not estimate the reductions in cost but actually generate new grammars for all instances of the operators and then calculate the reductions in cost. They also follow separate loops of applying a series of merge and combine operators, but we follow only one loop for both the operators.

#### Combine operator

This operator combines two or more non-terminals to form a new non-terminal. For example, if the non-terminals "DT ADJ NN" appear very often in the current grammar, then the cost (equivalently size) of the grammar can be reduced by introducing a new production *C*1 → *DT ADJ NN*, where *C*1 is a system generated non-terminal. Next, all the occurrences of *DT ADJ NN *on the RHS of the productions will be replaced by *C*1. As can be seen, this reduces the size of all those productions but at the same time adds a new production and a new non-terminal. In [3], the corresponding operator only combined two non-terminals at a time and could combine more than two non-terminals only upon multiple applications of the operator (for example, first combine DT and ADJ into C1 and then combine C1 and NN into C2). But we found this was less cost-effective in the search procedure than directly combining multiple non-terminals, hence we generalized the operator.

It may be noted that this operator only changes the cost of the grammar and not the cost of the derivation. This is so because in the derivations, the only change will be the application of the extra production (like *C*1 → *DT ADJ NN*), and since there is only one way to expand the new non-terminal *C*1, there is no need to encode it (i.e. |*P*(*C*1)| is 1, hence its log is zero in equation 4). It is also interesting to note that this operator does not increase the coverage of the grammar, i.e., the new grammar obtained after applying the *combine *operator will not be able to parse any new sentence that it could not parse before. The coverage does not decrease either.

The reduction in cost due to applying any instance of this operator can be estimated easily in terms of the number of non-terminals being combined and how many times they occur adjacently on the RHS of current productions in the grammar. Note that if the non-terminals do not appear adjacent enough number of times then this operator can, in fact, increase the cost.

#### Merge operator

This operator merges two non-terminals into one. For example, it may replace all instances of *NNP *and *NNS *non-terminals in the grammar by a new non-terminal *M*1. This operator is the same as in [3]; we did not generalize it to merging more than two non-terminals, because unlike the *combine *operator, it is combinatorially expensive to find the right combination of non-terminals to merge (for the *combine *operator, we describe this procedure in the next subsection).

The *merge *operator could eliminate some productions. For example, if there were two productions *NP *→ *DT NNP *and *NP *→ *DT NNS*, then upon merging *NNP *and *NNS *into *M*1, both the productions reduce to the same production *NP *→ *DT M*1. This not only reduces the cost of the grammar by reducing its size, but also reduces the |*P*(*NP*)| value (how many productions have *NP *on LHS) which results into a further decrease in the derivation cost (equation 4). However, if there were productions with *NNP *and *NNS *on the LHS, then their getting combined will make the cost of |*P*(*M*1)| equal to the sum of |*P*(*NNP*)| and |*P*(*NNS*)| and replacing *NNP *and *NNS *by *M*1 everywhere in the derivations will increase the cost of the derivations.

To estimate the reduction in cost due to applying any instance of this operator, one needs to estimate which productions will get merged (hence eliminated) and in how many other productions does the non-terminal on the LHS of these productions appear on LHS. In our implementation, we efficiently do this by maintaining data structures relating non-terminals and the productions they appear in, and relating the productions and the derivations they appear in. We are not describing those details here due to lack of space. As mentioned before, while the cost may decrease for some reasons, it could also increase for other reasons. Hence an application of an instance of this operator can also increase the overall cost.

It is important to mention that application of this operator can only increase the coverage of the grammar. For example, given productions *NNS *→ *apple*, *V B *→ *eat *and *V P *→ *V B NNP*, but not a production *V P *→ *V B NNS*, then "*eat apple*" cannot be parsed into into *V P*. However, merging *NNP *and *NNS *into *M*1 will result in new productions *M*1 → *apple *and *V P *→ *V B M*1 which will parse "*eat apple*" into *V P*. Hence this operator generalizes the grammar.

#### Search procedure

Our method follows a greedy search procedure to find the grammar which results in the minimum overall cost of the grammar and the derivations (equation 5). Given a set of unannotated training sentences, it starts with the trivial, overly specific, extreme type of grammar in which a production is included for each unique sentence in the training set, as mentioned before. Next, all applicable instances of both the *combine *and *merge *operators are considered and the reduction in cost upon applying them is estimated. The instance of the operator which results into the greatest cost reduction is then applied. This process continues iteratively until no instance of the operator results in any decrease in cost. The resultant grammar is then returned as the induced grammar.

In order to find all the applicable instances of the *combine *operator, all "n-grams" of the non-terminals on the RHS are considered (the maximum value of n was 4 in the experiments). There is no reason to consider an exponentially large number of every combination of non-terminals which do not even appear once in the grammar. However, in order to find all the applicable instances of the *merge *operator, there is no such simple way but to consider merging every two non-terminals in the grammar (it is not obvious that any other way will be significantly more efficient with regards to estimating the reductions in cost). The start symbol of the grammar is preserved and is not merged with any other symbol. Note that this search procedure is greedy and may only give an approximate solution which could be a local minima.

### Obtaining production probabilities

The method described in the previous subsections induces a grammar but does not give the probabilities associated with its productions. If there are multiple ways in which a sentence can be parsed using a grammar, then having probabilities associated with its productions provide a principled way to choose one parse over another in a probabilistic context-free grammar parsing setting [17]. In this subsection, we describe an augmentation to our method to obtain these probabilities using an instance of the expectation-maximization (EM) [8] algorithm. As an initialization step of this algorithm, the probabilities are first uniformly assigned to all the productions that expand a non-terminal so that they sum up to a total of one. For example, if there are four productions that expand a non-terminal, say *NP*, then all those four productions will be assigned an equal probability of 0.25. Next, using these probabilities, the training sentences are parsed and the most probable parse is obtained for each of them. In the implementation, we used a probabilistic version [18] of the well known Earley's parsing algorithm for context-free grammars [19]. In the following iteration, assuming as if these parses are the correct parses for the sentences, the method counts how many times a production is used in the parses and how many times its LHS non-terminal is expanded in them. The corresponding fraction is then assigned as the probability of that production, similar to the way probabilities are computed in a supervised parsing setting from sentences annotated with correct parses. Using these as the new probabilities, the entire process is repeated under a new iteration. Experimentally, we found that this process converges within five iterations. Instead of only choosing the most probable parse for every sentence in each iteration, we also experimented with choosing all parses for a sentence and counting fractional counts proportional to the probabilities of the parses. However, this did not make any big difference.

## Experiments

### Methodology

To create a dataset, we took the first 5000 sentences from the discharge summaries section of the Pittsburgh corpus [9] using Stanford CoreNLP's sentence segmentation utility. We ran MetaMap [14] on these sentences to get part-of-speech tags and UMLS semantic types of words and phrases. MetaMap appeared to run endlessly on some long sentences hence we restricted to sentences with maximum length 20 (i.e. all 5000 sentences were of maximum length 20). Since many UMLS semantic types seemed very fine grained, we chose only 27 of them which seemed relevant for clinical reports (these included "disease or syndrome", "finding", "body part, organ, or organ component", "pathologic function", "medical device" etc.). All the occurrences of these semantic types were substituted for the actual words and phrases in the sentence. Figure 1(a) shows an original sentence from the corpus and 1(b) shows the same sentence in which some words and phrases have been substituted by their UMLS semantic types. Figure 1(c) shows the part-of-speech tags of the words of the original sentence as obtained by MetaMap. Note that the part-of-speech tags that MetaMap outputs are not as fine-grained as are in the Penn treebank. Also note that the entire last phrase "ventricular assist device" is tagged as a single noun. Finally, the words which were not replaced by the chosen UMLS semantic types were replaced by their part-of-speech tags.

Figure 1(d) shows the original sentence from 1(a) with words and phrases replaced by part-of-speech tags and UMLS semantic types. We ran all our experiments on sentences transformed into this final form. We note that our experiments are obviously limited due to the accuracy of MetaMap in determining the correct part-of-speech tags and UMLS semantic types.

We separated 1000 sentences from the 5000 sentences and used them as test sentences to determine how well the induced grammar works on novel sentences. The rest were used as the training data to induce grammar. Out of these 1000 test sentences, we manually put correct parsing brackets on 100 sentences to test the performance of the parses obtained by the induced grammar. We are not aware of any annotated corpus of clinical report domain which we could have used to measure this performance.

## Results and discussion

**Figure 2:**
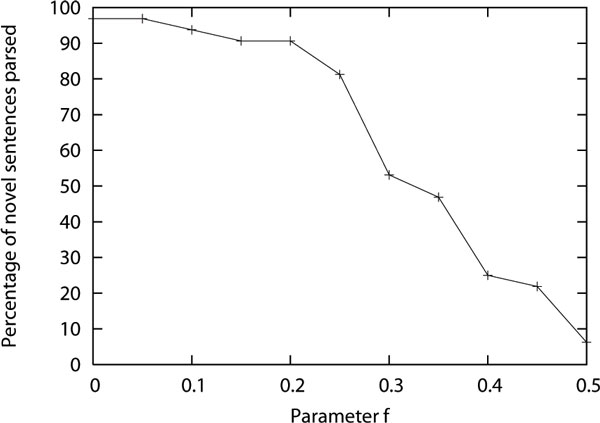
**Percentage of novel sentences parsed by different grammars induced by minimizing the total cost of the grammar and the derivations as the value of parameter f, which relatively weighs the two costs, is varied**.

We first show that by varying the parameter *f *of the total cost (equation 5), which weighs the relative contribution of the cost of grammar and the cost of derivations, the grammar induction method is capable of inducing a range of grammars, from very general ones to very restrictive ones. In this experiment, we only considered sentences which have at most 10 words for both training and test sentences (80% of the sentences in the corpus were of maximum length 10). We later show that performance decreases as we increase the maximum length of the sentence. We apply the method of inducing grammar on 4000 training sentences and measure how many novel sentences in the test data (i.e. which are not same as any of the training sentences) were parsable using the induced grammar. Since the grammar induction process starts with the grammar that can parse the training sentences and the grammar transformation operators never reduce its coverage, the induced grammar will always parse the sentences which are present in the training set. Out of the 791 sentences in the test data with maximum length of ten, 554 sentences overlapped with the training sentences (clinical reports have many repeated sentences). The remaining 237 sentences were novel. In Figure 2, we plot what percentage of these novel sentences the induced grammar was able to parse as we varied the *f *parameter from 0 to 0.5. The parses were obtained using Earley's context-free grammar parsing algorithm [19]. If *f *is more than 0.5, the induced grammar becomes so restrictive that it almost never parses any novel sentence. It can be seen that with smaller *f *value, the induction method tries to minimize the cost of the grammar more than the cost of the derivations and hence comes up with a small grammar that is very general and is able to parse almost any sentence. But with larger *f *values, the induction method tries to minimize the cost of the derivation more and comes up with a large grammar which is not very different from a production for every training sentence, and hence cannot parse many novel sentences. In this experiment we disallowed learning recursive productions by making sure a grammar transformation operator does not lead to a recursive production. We did so because we found that by allowing recursive productions, the percentage of novel parses go from near 100% down to near 0% with nothing in between. Recursions in productions drastically reduce the size of the grammar, hence the process otherwise prefers recursive productions which often leads to small overly general grammars (similar to an example given at the beginning of Subsection ).

**Figure 3:**
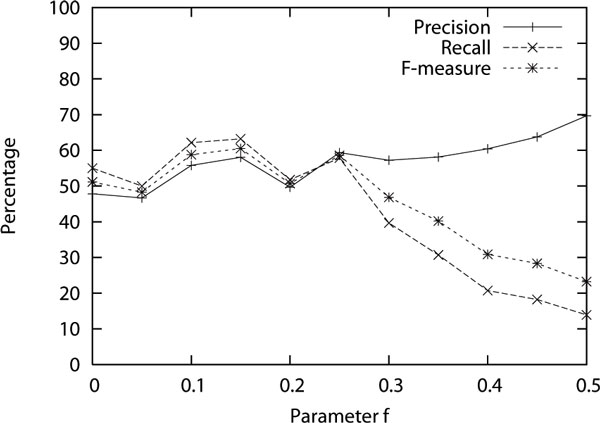
**Precision, recall and F-measure of the parsing brackets while varying the value of parameter f which relatively weighs the cost of the grammar and cost of the derivations during the grammar induction process**.

Please note that being able to parse a sentence does not necessarily mean that the parse is correct; we show this accuracy in Figure 3. Out of the 100 sentences that we manually annotated with correct parse brackets, 70 sentences were of maximum 10 words. We measured *precision*, how many of the brackets (start and end word) in the parses obtained by the induced grammar were present in the correct parses, *recall*, how many of the correct brackets were present in the obtained parses, and *F-measure*, the harmonic mean of precision and recall. We did not consider brackets containing one word and the entire sentence since they will be always correct. We measured these on novel sentences as well as the sentences which are also present in the training data because the system is not given the correct parses of the training sentences and so it makes sense to also measure the parsing performance on them. We measure the performance of bracketing because labeling accuracy cannot be measured since the system can only come-up with system-generated labels (like *M*1, *C*1 etc.). As it can be seen from Figure 3, the precision increases with higher values of *f *(more restrictive grammars) but the recall overall decreases. The F-measure is found to be maximum in between (60.5% at 0.15 as the value of the parameter *f*).

**Figure 4:**
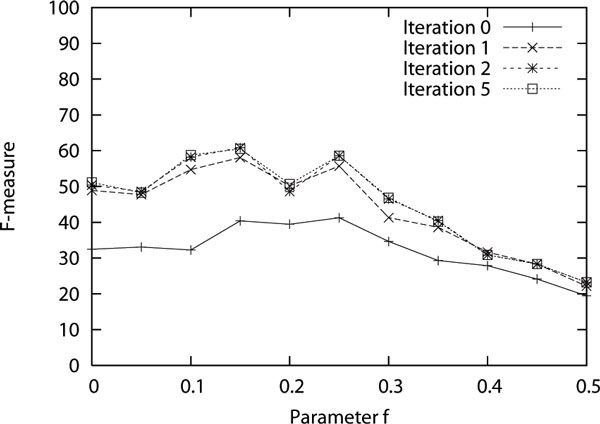
**F-measure of the parsing brackets with increasing number of iterations of the EM algorithm for estimating probabilities of the productions of the induced grammar**.

Earlier, in the above results, the F-measure had a maximum around 45%. Upon error analysis, we noticed that most of the errors were because the induced grammar would incorrectly pair subject and verb instead of the traditional way of pairing verb and object. There were also errors of pairing nouns followed by a preposition. Similar errors have been reported previously [6]. These errors could either be because the search for an optimum grammar is only approximate or it could be because these are in fact reasonable alternate parses. Nevertheless, in order to see its effect we introduced hard rules in the system to never let the part-of-speech tags of verb, det, prep, aux and conj be the second or later RHS term in any production. This increased the F-measure. But as can be seen from the wide range of F-measures in Figure 3, these rules alone are not sufficient to guarantee good performance. In future, perhaps these biases could be learned from a small amount of supervised data in a semi-supervised grammar induction setting. In the results shown in Figure 3, five iterations of the EM algorithm were performed to obtain the probabilities of the productions. Figure 4 shows how this performance changes with increasing number of iterations. Only the F-measure is shown for simplicity. The curve with iteration zero shows the performance when no probability is assigned to the productions and simply the first parse is returned when multiple parses are possible. The curve with one iteration shows the performance when uniform probabilities are assigned to the production under the initialization step. The remaining curves show the performances after subsequent iterations. It can be seen that a huge improvement is obtained once probabilities are used even when they are simply uniform. The performance shows a small improvement with a few more iterations but converges well within five iterations.

**Figure 5:**
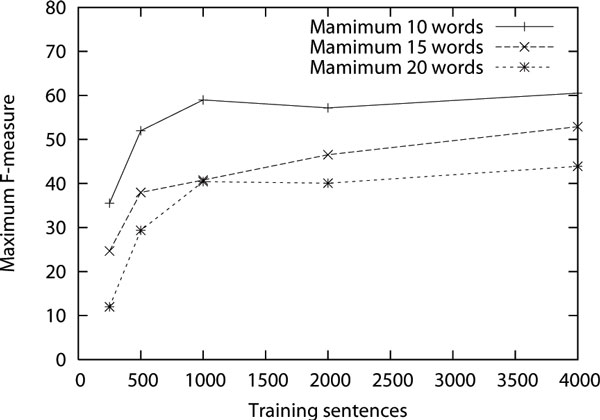
**F-measure of the parsing brackets with different amounts of training data and different maximum length of sentences**.

Finally, we present results on increasing the size of the training data and increasing the maximum length of the sentences (for both training and test sets) in Figure 5. The performance was measured on the same bracket-annotated corpus of 100 sentences all of which were of maximum length 20, 70 of these sentences had maximum length of 10 and 92 had maximum length of 15. For each point in the graph, we are showing the maximum F-measure that was found upon varying the parameter *f*. As can be seen, the accuracy decreases with longer lengths of sentences. It is interesting to note that the performance seems to have plateaued with the number of training examples.

## Future work

An obvious future task is to apply this approach to other genres of clinical report present in the Pittsburgh corpus. We, in fact, already did this, except for manually creating a corresponding bracket-annotated corpus for measuring parsing performance. Also, a bigger annotated corpus for evaluating the current results on discharge summaries genre is desirable. Another avenue of future work is to improve the search procedure for finding the optimum grammar. One way would be to do a beam search. Besides using the UMLS semantic types, in future, one may decide additional semantic types which could help in some application, for example, a negation class of words, a class of words representing patients etc. Currently the method first induces the grammar and then estimates the probabilities of its productions from the same data. An interesting possibility for future work will be to integrate the two steps so that the probabilities are computed and employed even during the grammar induction process. This will be a more elegant method and will likely lead to an improvement in the parsing performance.

## Conclusions

Unsupervised parsing is particularly suitable for clinical domains because it does not require expensive annotation effort to adapt to different genres and styles of clinical reporting. We presented an unsupervised approach for inducing grammar for clinical report sublanguage in terms of part-of-speech tags and UMLS semantic types. We showed that using the cost-reduction principle, the approach is capable of learning a range of grammars from very specific to very general and achieves the best parsing performance in between.

## Competing interests

The author declares that they have no competing interests.
